# Cytosolic Innate Immune Sensing and Signaling upon Infection

**DOI:** 10.3389/fmicb.2016.00313

**Published:** 2016-03-14

**Authors:** Lilliana Radoshevich, Olivier Dussurget

**Affiliations:** ^1^Unité des Interactions Bactéries-Cellules, Institut PasteurParis, France; ^2^Institut National de la Santé et de la Recherche Médicale, U604Paris, France; ^3^Institut National de la Recherche Agronomique, USC2020Paris, France; ^4^Cellule Pasteur, Université Paris Diderot, Sorbonne Paris CitéParis, France

**Keywords:** immunity, pathogen, STING, MAVS, DNA, RNA, interferon

## Abstract

Cytosolic sensing of pathogens is essential to a productive immune response. Recent reports have emphasized the importance of signaling platforms emanating from organelles and cytosolic sensors, particularly during the response to intracellular pathogens. Here, we highlight recent discoveries identifying the key mediators of nucleic acid and cyclic nucleotide sensing and discuss their importance in host defense. This review will also cover strategies evolved by pathogens to manipulate these pathways.

## Introduction

Surveillance of the cytosol by host pathogen recognition receptors is essential to activation of the innate immune response and pathogen clearance. It has long been appreciated that cell surface receptors can identify pathogen-associated molecular patterns to initiate the innate immune response. More recently, a concept is emerging in the context of intracellular sensing of pathogens. In particular, if molecules specific to pathogens or shared by pathogens and host are located in an inappropriate cellular compartment (i.e., DNA in the cytosol), this triggers innate immune signaling. Subsequent signaling downstream of pathogen recognition is also compartmentalized to organelle-based signaling platforms on the surface of the endoplasmic reticulum (ER), the mitochondria and peroxisomes. In addition, in the ongoing arms race between pathogen and host, the various nodes of pathogen surveillance and signaling are frequently targeted by a variety of bacterial and viral effectors. In this review, we have chosen to focus on cytosolic sensors of DNA, RNA, and cyclic dinucleotides and their subversion by bacterial and viral pathogens.

## DNA Cytosolic Surveillance Pathways

### DNA Sensing through STING

In 2006 cytosolic DNA from the bacterial pathogen *Listeria monocytogenes* was first reported to lead to a type I interferon (IFN) response (Stetson and Medzhitov, 2006). More recently the field of cytosolic DNA and cyclic dinucleotide (CDN) sensing has made significant conceptual and mechanistic advances. Initially, STING (Stimulator of Interferon Genes) was cloned and characterized as an ER-localized transmembrane protein that was an essential signaling adaptor to IFN production (Ishikawa and Barber, 2008). Subsequent work identified the minimal DNA motifs required for IFN production and the effect of STING on *in vivo* infection and T cell mediated immunity (Ishikawa et al., 2009). A parallel breakthrough occurred when several groups determined that cytosolic CDNs – small nucleotides that are unique to bacteria – from a variety of pathogens provoke an IFN response (Karaolis et al., 2007a,b; Mcwhirter et al., 2009; Woodward et al., 2010). STING’s central role in this process was highlighted through a forward genetic screen which identified a mutant of STING that was unresponsive to CDNs and through a second study which proved a direct biochemical interaction between STING and cyclic-diguanylate (c-di-GMP; Burdette et al., 2011; Sauer et al., 2011). Most intriguingly, mutants of STING that could bind CDNs but did not activate downstream signaling were still capable of responding to cytosolic DNA. These data as well as the observation that STING expression alone in 293T cells could not complement the deficiency of this cell line in sensing cytosolic DNA suggested a missing DNA sensor (Burdette et al., 2011). One report posited that STING could directly bind single stranded or double stranded DNA, however, the same region of STING was important for CDN binding (Abe et al., 2013), data that is difficult to reconcile with earlier studies that highlighted the capacity of mutant STING (that could not signal following CDN binding) to induce IFN downstream of cytosolic DNA.

**FIGURE 1:**
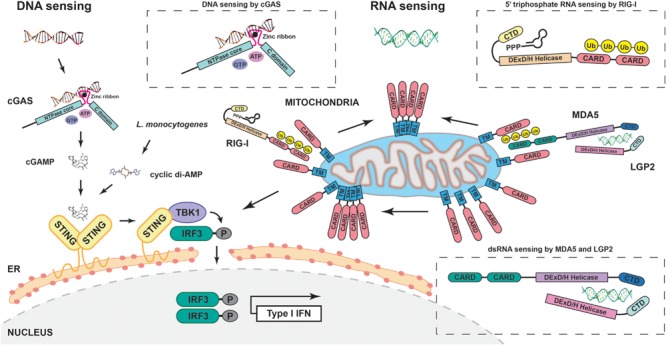
**Schematic representation of major cytosolic DNA and RNA sensing pathways.** Cytosolic DNA is sensed by cGAS leading to the production of cGAMP and subsequent STING/TBK1/IRF3 signaling, and type I IFN production; bacterial cyclic dinucleotides (like *L. monocytogenes* cyclic di-AMP) also activate STING. 5′ triphosphate RNA and double stranded RNA are sensed by RIG-I and either MDA-5 alone or MDA-5/LGP2. This leads to polymerization of mitochondrial MAVS and also activates TBK1 and IRF3.

Over the past two years several groups have identified a cyclic dinucleotide eukaryotic second messenger, 2′3′-cyclic guanosine monophosphate–adenosine monophosphate (cGAMP), which led to the discovery of the cyclic GMP-AMP synthase (cGAS) as the missing cytosolic DNA sensor linking STING activity to IFN production (**Figure 1**). Using a cell free system, cGAMP was generated and identified following exposure of the cytosol to DNA or infection with a DNA virus (Wu et al., 2013b). Interestingly, this second messenger has a unique structure which differentiates it from bacterial CDNs and potentially renders it less susceptible to phosphodiesterases (Ablasser et al., 2013; Diner et al., 2013; Gao et al., 2013a; Zhang et al., 2013). This was a major conceptual leap since structurally cGAS could directly interact with DNA and then through a unique enzymatic function, it could generate a second messenger capable of activating STING (Sun et al., 2013). In addition, cGAS deletion phenocopies STING deletion with regards to IFN production following activation, a property which all of the other previously described DNA sensors lack. The initial structures of STING bound to c-di-GMP were rather perplexing, as binding did not lead to a conformational change (Cai et al., 2014). Binding of the cGAS product, cGAMP, on the other hand leads to a conformational change of STING (Gao et al., 2013b; Zhang et al., 2013).

Other cytoplasmic DNA sensors involved in innate immune responses are the DNA-dependent protein kinase (DNA-PK) complex and the MRN complex. The two complexes are both sensors and effectors of the DNA damage response (DDR). DNA-PK is a heterotrimeric complex composed of the heterodimer Ku70/Ku80 and the catalytic subunit DNA-PKcs. The MRN complex consists of meiotic recombination 11 (Mre11), which has been linked to STING activation, radiation-sensitive 50 (Rad50) and Nijmegen breakage syndrome 1 (Nbs1) proteins. DNA sensing by DNA-PK and MRN is critical for innate immune responses and DDR, inhibiting viral DNA replication.

A few other proteins, including DNA-dependent activator of IFN-regulatory factors (DAI) and interferon-inducible protein 16 (IFI16), have been previously suggested to be sensors of DNA. However, consensus on how and whether they act upstream of STING has not been reached. In addition, none of these proteins has a comparably profound effect on IFN signaling as cGAS when deleted (Paludan and Bowie, 2013). Therefore, understanding how the DNA-sensing pathways intersect and complement each other in various cell types or tissues is an important future direction.

### Cellular Responses

Following STING activation on the surface of the ER by CDNs, STING interacts with the TNFR-associated NF-κB kinase (TANK)-binding kinase 1 (TBK1) and relocalizes to cytoplasmic puncta. These puncta are also microtubule-associated protein 1 light chain 3 (LC3), autophagy-related gene 9 (ATG9) and p62 positive (Saitoh et al., 2009). TBK1 can subsequently phosphorylate interferon regulatory factor 3 (IRF3) leading to IFN production. Chen et al. (2011) have recently highlighted a key role played by signal transducer and activator of transcription 6 (STAT6) in this process. Interestingly, STAT6 becomes phosphorylated and active following viral infection in a Janus kinase (JAK)-independent manner that requires STING and leads to a different subset of STAT6 target genes than canonical STAT6 activation (Chen et al., 2011). Several recent papers have highlighted early IFN-independent induction of interferon-stimulated genes (ISGs) following viral or bacterial infection. The cytosolic 3′ repair exonuclease 1 (TREX1) degrades excess cytosolic DNA and mutations in this gene are associated with the autoimmune disease Aicardi–Goutières syndrome (Stetson et al., 2008). Hasan et al. (2013) took advantage of TREX1-deficient cells to discover a cell-intrinsic IFN-independent cytosolic surveillance pathway in response to viral infection. While IFN production was the same for RNA viruses in wild-type or TREX1-deficient cells, viral load was much lower due to a baseline increase in ISGs. Interestingly, this increase was dependent on STING/TBK1/IRF3 and IRF7 and also led to increased lysososomal biogenesis (Hasan et al., 2013). Our own group independently found an IFN-independent induction of ISG15 upon infection of non-phagocytic cells with *L. monocytogenes*. This IFN-independent induction required STING/TBK1/IRF3 and IRF7 and interestingly could be recapitulated by *Listeria* genomic DNA but not bacterial cyclic di-AMP (Radoshevich et al., 2015). An exciting future direction will be to determine varying phenotypic outcomes based on the subset of ISGs that are induced during a variety of infections. Another critical theme that has emerged from several recent studies is the link between STING/TBK1/IRF3 signaling and autophagy. In particular, one surprising finding is that the membrane-limited pathogen *Mycobacterium tuberculosis* activates the cytosolic surveillance pathway to a comparable degree as the intracellular cytosolic pathogen *L. monocytogenes* (Manzanillo et al., 2012). These data imply that *M. tuberculosis* DNA is being sensed by cGAS, which in turn activates the STING/TBK1/IRF3 signaling axis and anti-bacterial autophagy to help with pathogen clearance. In fact, three papers have recently demonstrated the crucial role of cGAS during *M. tuberculosis* infection (Collins et al., 2015; Wassermann et al., 2015; Watson et al., 2015). Future questions of interest to the field will be to determine whether the bacterium is secreting its own DNA or lysed bacteria are the source of the DNA signal. In addition it will be informative to compare and contrast signaling outcomes from eukaryotic versus bacterial CDNs in terms of which ISGs are induced and whether this has a functional outcome on the adaptive immune response. In the case of *Listeria* for example cyclic di-AMP secretion negatively affects T-cell mediated immunity but it would be interesting to dissect the role of eukaryotic cGAMP in this context (Archer et al., 2014).

## RNA Cytosolic Surveillance Pathways

### RNA Sensing through MAVS

Since viral infection generates pathogen-associated molecular patterns such as double stranded RNA, the field of RNA sensing made earlier advances than the field of DNA sensing following the search for cytosolic sensors of these pathogen-specific molecules. Several landmark papers elucidated a role for TBK1 and IκB kinase-*i* (IKK-*i*) as kinases critical for phosphorylation and activation of IRF3 and IRF7 both *in vitro* and *in vivo* (Fitzgerald et al., 2003; Sharma et al., 2003; Hemmi et al., 2004; Mcwhirter et al., 2004; Perry et al., 2004). Subsequently, two groups demonstrated the ability of known proteins, retinoic acid inducible gene-I (RIG-I) and melanoma differentiation-associated gene-5 (MDA-5), to bind dsRNA (**Figure 1**). RIG-I was identified as a protein that could lead to IRF activity in the presence of dsRNA in a large scale cDNA screen (Yoneyama et al., 2004). Further experiments showed that this activity followed direct dsRNA binding by the cytosolic protein RIG-I. Interestingly, a mutant of RIG-I solely expressing its caspase-recruitment domain CARD was sufficient to spontaneously activate the IFN-producing activity. At the time this suggested, and the authors predicted, that this domain interacts with an as yet unidentified downstream molecule, a hypothesis proven shortly thereafter. Parallel work demonstrated that MDA-5 was a second direct sensor of viral RNA. As for RIG-I, ectopic expression of MDA-5 was sufficient to induce IFN signaling (Andrejeva et al., 2004). RIG-I and MDA-5 play complementary but separate roles in the response to viral infection. More specifically, genetic ablation of MDA-5 was shown to block the response to poly(I:C) whereas loss of RIG-I abrogated sensing of *in vitro* transcribed dsRNA (Kato et al., 2006). Several groups went on to identify the ligand for RIG-I as uncapped 5′ triphosphate RNA and to biochemically characterize the interaction (Hornung et al., 2006; Pichlmair et al., 2006; Schlee et al., 2009). Interestingly, the 5′ triphosphate RNA must form internal base pairs with a blunt end to be stimulatory (Schmidt et al., 2009). Finally, the structure of RIG-I in complex with dsRNA was solved in 2011 (Jiang et al., 2011; Kowalinski et al., 2011). In the case of the ligand for MDA-5, 2′-*O*-methylation normally occurs in the cap of eukaryotic RNA molecules and is mimicked by viruses. Deletion of enzymes required for this modification leads to increased IFN production dependent on MDA-5 sensing, suggesting that RNA that lacks this modification is the MDA-5 ligand (Züst et al., 2011). The structure of this interaction was finally solved in 2013 (Wu et al., 2013a), shedding light on the physical basis for distinct ligand sensing mediated by homologous regions of RIG-I and MDA-5. LGP2 (Laboratory of genetics and physiology 2) is the third member of the RIG-I like receptor (RLRs) family however, much less is known about its mechanism of action. It too can bind dsRNA, but seems to repress RIG-I or compete with it, while it can activate MDA-5 (Li et al., 2009; Pippig et al., 2009). A recent hypothesis is that LGP2 synergizes with MDA-5, since LGP2 has superior dsRNA binding capacity but reduced signaling efficacy whereas MDA-5 is a relatively weaker binder of dsRNA but signals much more efficiently than LGP2 (Bruns et al., 2014). Thus, a pertinent future direction would be to dissect the interplay between the three molecules, potentially using a genetic deletion model.

Following the aforementioned prediction of a downstream signaling effector of RIG-I, a flurry of activity led to the cloning and identification of the MAVS (mitochondrial-antiviral signaling) protein by four separate groups. Two of the groups took a bioinformatics approach mining the genome for CARD domains that resembled that of MDA-5 and RIG-I (Meylan et al., 2005; Seth et al., 2005). The other two groups identified MAVS using unbiased high-throughput screens for IFN production (Kawai et al., 2005; Xu et al., 2005). MAVS has a CARD domain, a leucine rich repeat and a transmembrane domain, which tethers the protein to the mitochondrial membrane (Seth et al., 2005). MAVS can physically interact with RIG-I and is epistatic to TBK1 and IKK-*i.* Most importantly, MAVS plays a functional role in the immune response to viruses, i.e., its depletion leads to increased viral load and its overexpression reduces viral load. More recently, the molecular details of the interactions between RIG-I, MAVS, and viral RNA were elucidated using a novel cell-free system that incorporates isolated mitochondria (Zeng et al., 2010). Other work had previously demonstrated the role of K63-linked ubiquitin modification in RIG-I signaling (Zeng et al., 2009). More specifically, critical signaling molecules in innate immune pathways, such as RIG-I, become modified by polyubiquitin chains linked via the K63 residue of ubiquitin (Gack et al., 2007). Rather than leading to degradation of the target protein, this linkage enhances the signaling capacity of the target. However, using the cell free system, the role of free polyubiquitin K63 chains emerged as a requirement in RIG-I/MAVS signaling (Zeng et al., 2010). Another seminal paper from the Chen lab put forth the concept that MAVS activation accompanies a conformational change in and polymerization of the protein which travels across the mitochondria in waves, reminiscent of a prion-type conversion (Hou et al., 2011). While the cell-free system has illuminated a number of novel concepts in RIG-I signaling, it will be important to validate these findings within cells and potentially *in vivo* as well. The clustering and conversion of MAVS also begs the question whether or not it can be restored to its pre-viral state or must it be removed and how does this affect mitochondrial fitness, morphology and clearance?

### Cellular Responses

Similar to the activation of STING-mediated signaling, clustering of MAVS leads to interferon production following activation of IRF3 and IRF7 and NF-κB activation, helping cells to establish an anti-viral state. While the vast majority of MAVS is anchored in the mitochondrial outer membrane, a fascinating paper identified that MAVS can also be peroxisomal (Dixit et al., 2010). Interestingly, this affects the downstream signaling response. Peroxisomal MAVS leads to an early interferon-independent induction of ISGs that requires IRF1, whereas mitochondrial MAVS contributes to the canonical type I IFN response. Subsequent work determined that in response to a number of viruses and at least one intracellular bacterium, peroxisomal MAVS signaling led to the induction of type III interferon (IFN-λ) and that this signaling was downstream of RIG-I sensing (Odendall et al., 2014). Interestingly, 5′ triphosphate RNA from bacterial pathogens such as *L. monocytogenes* can be sensed by RIG-I leading to IFN production in non-phagocytic cells (Abdullah et al., 2012; Hagmann et al., 2013). It is possible that the bacteria actively secrete these RNA molecules as a secretion deficient-mutant of the pathogen elicits a dampened immune response and 5′ triphosphate RNA is detectable at early time points of infection in the cytosol. These studies raise the question of whether early detection of pathogenic RNA affects T cell-mediated immunity as bacterially produced cyclic di-AMP does.

## Microbial Strategies for Subversion of Cytosolic Surveillance Pathways

### Microbial Strategies for Subversion of DNA Cytosolic Surveillance Pathways

Since STING is such a critical protein for innate immune sensing, it and its downstream signaling components are strategic targets of a number of pathogens. *Shigella flexneri* type III effector invasion plasmid antigen J (IpaJ) is a cysteine protease that cleaves the *N*-myristoylated glycine of lipidated ADP ribosylation factor (ARF)-family GTPases, disrupts the Golgi apparatus, thereby inhibiting host protein secretion (Burnaevskiy et al., 2014). Recently, it has been shown that IpaJ antagonizes STING-mediated IFNβ activation by blocking STING translocation from the ER to ER-Golgi intermediate compartments in mouse embryonic fibroblasts (Dobbs et al., 2015). This inhibition of immune detection is important for *Shigella* pathogenesis (Burnaevskiy et al., 2014). In contrast, other intracellular pathogens activate STING to inhibit the T cell-mediated immune response. For example, *L. monocytogenes* secretes c-di-AMP to activate STING, leading to production of IFNβ and inhibition of cell-mediated immunity (Woodward et al., 2010; Archer et al., 2014). Another trigger of IFNβ expression is *L. monocytogenes* DNA, which operates through IFI16, cGAS and STING in human macrophages (Hansen et al., 2014). Similarly, *M. tuberculosis* DNA, possibly secreted through the type VII secretion system ESX-1, associates with cGAS to stimulate a type I interferon response, which favors bacterial pathogenesis and disease progression (Wassermann et al., 2015).

Likewise viral DNA is recognized by STING and STING-dependent cytosolic sensors and triggers antiviral responses. Like bacterial pathogens, viruses have evolved sophisticated mechanisms to manipulate host DNA sensing. In order to evade the type I interferon response, the dengue virus (DENV) expresses the NS2B3 protease complex, which specifically cleaves STING in human dendritic cells (Aguirre et al., 2012). Interestingly, DENV NS2B3 inhibits type I interferon production in human but not in mouse cells, since the proteolytic complex does not degrade murine STING. Hence, STING restricts DENV replication in mouse cells. Several viral proteins can antagonize STING-dependent DNA sensing. Kaposi’s sarcoma-associated herpesvirus ORF52 protein directly inhibits cGAS enzymatic activity (Wu et al., 2015). Oncoproteins E7 from human papillomavirus and E1A from adenovirus bind to STING and antagonize the cGAS-STING pathway, preventing the antiviral response (Lau et al., 2015). Human cytomegalovirus tegument protein pUL83 binds IFI16 pyrin domain, blocking its oligomerization upon DNA sensing, preventing the expression of antiviral cytokines (Li et al., 2013). Herpes simplex virus-1 (HSV-1) ICP0 E3 ubiquitin ligase targets nuclear IFI16 for degradation, inhibiting nuclear innate immune sensing, cytoplasmic STING activation and antiviral responses (Orzalli et al., 2012). Murine cytomegalovirus protein M45 inhibits signaling mediated through DAI, which is required for DNA sensing and type I interferon production in some cells (Rebsamen et al., 2009).

Cytosolic foreign DNA not only activates STING-mediated innate immune responses, but it is also able to stimulate inflammasome activation, leading to IL-1β and IL-18 maturation and secretion. As such, some pathogens antagonize inflammasome signaling pathways. *M. tuberculosis*, but not non-virulent *Mycobacterium smegmatis*, inhibits AIM2 (absent in melanoma 2) inflammasome activation (Shah et al., 2013). While the *M. tuberculosis* AIM2 inhibitor is as yet unknown, it seems to be secreted by the ESX-1 secretion system.

The importance of inhibiting the inflammasome to promote viral infection has been demonstrated by revealing the critical role of the Poxvirus M13L immunomodulatory protein in infection of monocytes and lymphocytes and in disease (Johnston et al., 2005). Poxvirus M13L is a member of the pyrin domain-containing superfamily of proteins. It interacts with host pyrin domain protein ASC-1 (apoptosis-associated speck-like protein containing CARD-1), inhibiting caspase-1 activation in monocytes, thereby disrupting the intracellular pathways leading to IL-1β processing and secretion.

Other important DNA sensors such as DNA-PK and MRN are also targets of viral antagonists. Peters et al. (2013) have shown that vaccinia virus inhibits the innate immune response of infected fibroblasts by expressing the C16 protein. C16 directly binds to the heterodimer Ku70/Ku80, impairing DNA-PK binding to DNA and downstream signaling cascades. Adenovirus type 5 (Ad5) antagonizes the MRN-dependent DDR by two mechanisms. First, it triggers MRN proteasomal degradation upon ubiquitination by an E3 ligase complex composed of Ad5 E1B-55K and E4-ORF6 proteins associated with host proteins (Stracker et al., 2002; Weitzman and Ornelles, 2005). Second, it inhibits MRN activity by expressing E4-ORF3. Ad5 E4-ORF3 recruits nuclear proteins, such as promyelocytic leukemia (PML) and PML nuclear body-associated proteins, to sequester MRN proteins in the nucleus of infected cells (Stracker et al., 2002; Weitzman and Ornelles, 2005). These distinct mechanisms induce efficient inactivation of cellular DDR and antiviral defense.

### Microbial Strategies for Subversion of RNA Cytosolic Surveillance Pathways

Because cytoplasmic RNA sensors RIG-I, MDA-5 and LGP2 confer resistance to intracellular pathogens, many viruses have evolved strategies to control the RLRs. Some viruses are able to suppress RIG-I RNA sensing step. Indeed, both Marburg virus and Ebola virus VP35 proteins and severe acute respiratory syndrome coronavirus (SARS-CoV) nucleocapsid protein N antagonize RIG-I signaling by binding and masking dsRNA (Cárdenas et al., 2006; Lu et al., 2011; Bale et al., 2012, 2013). Of note, Ebola virus VP35 protein also binds dsRNA and inhibits the antiviral activity of the dsRNA-dependent protein kinase R (Feng et al., 2007). The vaccinia virus E3 protein binds to poly(A-U) RNA generated from viral dsDNA by RNA polymerase III, preventing RIG-I activation (Valentine and Smith, 2010). DENV is able to elude RIG-I recognition, possibly by formation of convoluted membranes tightly associated with the NS4A viral protein and convoluted-membrane vesicles containing dsRNA (Welsch et al., 2009). Some negative-stranded RNA viruses, such as Hantaan virus, Crimean–Congo hemorrhagic fever virus and Borna disease virus, cleave the 5′-triphosphate group of their genome, which therefore becomes invisible to RIG-I (Habjan et al., 2008). Arenaviruses 3′-5′ exoribonucleases, such as Lassa fever virus nucleocapsid protein NP, process viral dsRNA to prevent its recognition by RLRs (Jiang et al., 2013). Arenaviruses also produce decoys, i.e., short 5′-ppp dsRNA containing an overhanging GTP nucleotide, which trap RIG-I in inactive dsRNA complexes (Marq et al., 2011).

Some viral proteins bind to RLRs and inhibit their downstream signaling pathways, dampening the production of type I interferon. For example, influenza virus non-structural protein 1 (NS1) binds to RIG-I containing complexes and blocks RIG-I activation (Pichlmair et al., 2006). HSV-1 RNA binding tegument protein US11 binds to RIG-I and MDA-5 to antagonize the IFNβ pathway and promote pathogenesis (Xing et al., 2012). Z proteins from New World arenaviruses Guanarito virus, Junin virus, Machupo virus and Sabia virus, but not Old World arenavirus lymphocytic choriomeningitis virus or Lassa virus, bind to RIG-I and prevent the IFNβ response (Fan et al., 2010). The V proteins of paramyxoviruses bind MDA-5 and block its activity (Andrejeva et al., 2004; Motz et al., 2013). The V proteins of Nipah virus and Hendra virus bind LGP2 and induce the formation of stable LGP2/RIG-I complexes unable to recognize dsRNA (Parisien et al., 2009; Childs et al., 2012).

Other viral proteins abrogate RIG-I ubiquitination which has been shown to be critical for its antiviral activity (Gack et al., 2007). Influenza A virus NS1 specifically blocks multimerization of the TRIM25 ligase, thereby inhibiting RIG-I ubiquitination (Gack et al., 2009). Hepatitis C virus NS3-4A proteases target the Riplet ubiquitin ligase which is required for TRIM25-mediated activation of RIG-I (Oshiumi et al., 2013). Kaposi’s sarcoma associated herpes virus ORF64 tegument protein is a deubiquitinase, which inhibits RIG-I activation by suppressing its ubiquitination and reduces interferon signaling (Inn et al., 2011). A similar mechanism of immune escape has been evolved by arterivirus and nairovirus which express ovarian tumor domain-containing deubiquitinases targeting RIG-I (Frias-Staheli et al., 2007; van Kasteren et al., 2012), and by hepatitis E virus which expresses the ORF1 protein bearing a papain-like cystein protease domain responsible for RIG-I deubiquitination (Nan et al., 2014).

Another mechanism of RLR subversion is the inhibition of RNA-sensor dephosphorylation, which is necessary for their activation. Measles virus can suppress RIG-I and MDA-5 activation by binding to DC-SIGN (dendritic cell-specific intercellular adhesion molecule-3-grabbing non-integrin), which triggers activation of the kinase Raf-1 (Rapidly accelerated fibrosarcoma-1), association of protein phosphatase 1 (PP1) inhibitor I-1 with GADD34-PP1 phosphatases and ultimately inhibition of RIG-I and MDA-5 dephosphorylation (Mesman et al., 2014).

Not surprisingly, MAVS is also targeted by several viral proteins to antagonize type I interferon responses. SARS-CoV ORF3b, ORF6, NP and M proteins and influenza A virus NS1, PB1, PB2, PA, and PB1-F2 proteins inhibit production of type I interferon, in most cases through direct interaction with MAVS (Kopecky-Bromberg et al., 2007; Freundt et al., 2009; Siu et al., 2009; Iwai et al., 2010; Varga et al., 2012; Zinzula and Tramontano, 2013). Other viruses abrogate MAVS signaling indirectly. For example, vaccinia virus K7 protein and hepatitis C virus core protein interact with DEAD box protein 3 (DDX3) to interfere with MAVS signaling (Schröder et al., 2008; Oshiumi et al., 2010).

## Conclusion and Perspectives

The field of cytosolic innate immunity has progressed tremendously in the past decade. A multiplicity of DNA and RNA cytosolic sensors and signaling pathways has been discovered, revealing an unexpected complexity of the response to nucleic acids of pathogens. Key questions remain to be answered. What is the role of each of these pathways in different cell types and tissues? What is their respective importance in clearance of infection in different animal species, including humans? On the pathogen side, a plethora of immune escape mechanisms developed by viruses have been unraveled over the years. Fewer bacterial determinants inhibiting cytosolic immune sensing have been clearly elucidated so far and future work will surely lead to the identification of many more. Microbial proteins targeting cytosolic sensing have been repeatedly shown to be virulence factors important for pathogenesis. Further characterization of these factors could lead to the development new preventive or therapeutic antimicrobial strategies.

## Author Contributions

All authors listed, have made substantial, direct and intellectual contribution to the work, and approved it for publication.

## Conflict of Interest Statement

The authors declare that the research was conducted in the absence of any commercial or financial relationships that could be construed as a potential conflict of interest.
